# Avoidable deaths in Sweden, 1997–2018: temporal trend and the contribution to the gender gap in life expectancy

**DOI:** 10.1186/s12889-021-10567-5

**Published:** 2021-03-17

**Authors:** Ali Kiadaliri

**Affiliations:** 1 Clinical Epidemiology Unit, Department of Clinical Sciences Lund, Orthopaedics, Lund University, Skåne University Hospital, Remissgatan 4, SE-221 85 Lund, Sweden; 2 Centre for Economic Demography, Lund University, Lund, Sweden

**Keywords:** Avoidable, Amenable, Gender gap, Life expectancy, Mortality, Preventable, Sweden, Temporal trend

## Abstract

**Background:**

Avoidable mortality is considered as a potential indicator of the influences of public health policies and healthcare quality on population health. This study aimed to examine the trend in avoidable mortality and its influence on rising life expectancy (LE) and declining gender gap in LE (GGLE) in Sweden.

**Methods:**

We extracted data on causes of death by age, sex, and year from national registry from 1997 to 2018. The UK Office for National Statistics definition was used to divide causes of death into five mutually exclusive categories: amenable, preventable, amenable & preventable, ischemic heart disease (IHD), and non-avoidable causes. We applied Joinpoint regression to analyse temporal trends in age-standardized mortality rates. The Arriaga method was applied to decompose changes in LE and GGLE by age group and causes of death.

**Results:**

Average annual reductions in avoidable vs. non-avoidable mortality were 2.6% (95% CI:2.5, 2.7) vs. 1.4% (95% CI:1.3, 1.5) in men, and 1.6% (95% CI:1.4, 1.9) vs. 0.9% (95% CI:0.7, 1.0) in women over the study period. LE in men rose by 4.1 years between 1997 and 2018 (from 72.8 to 76.9 years), of which 2.4 years (59.3%) were attributable to reductions in avoidable mortality. Corresponding LE gain was 2.3 years in women (from 78.0 in 1997 to 80.3 in 2018) and avoidable mortality accounted for 1.0 year (45.6%) of this gain. Between 1997 and 2018, the GGLE narrowed by 1.9 years, of which 1.4 years (77.7%) were attributable to avoidable causes. Among avoidable causes, while preventable causes had the largest contribution to the GGLE, IHD had the greatest contributions to LE gains and the narrowing GGLE.

**Conclusions:**

Our findings showed that avoidable causes had a substantial contribution to gain in LE with more profound gain in men than in women, resulting in narrowing the GGLE. Lower pace of reductions in preventable than amenable mortality highlights the need for improving the effectiveness of inter-sectoral health policies aimed at behavioural changes.

**Supplementary Information:**

The online version contains supplementary material available at 10.1186/s12889-021-10567-5.

## Background

Life expectancy (LE) is an important summary measure of population health. It represents the average number of years a person can expect to live given the current age-specific mortality rates. Around the world, women live longer than men, even though the gender gap in LE (GGLE) has been declining in many developed countries since 1980s [1]. In Sweden, the GGLE has decreased from 5.2 years in 1997 to 3.6 years in 2014 [2]. Biological (e.g. genetic and hormones), behavioural (e.g. life style), and socioeconomic (e.g. social roles and occupational hazards) factors have been proposed as possible explanations for this gender disparity [3–5]. Moreover, the narrowing GGLE has been attributed to declining gender differences in some of these factors including rising smoking among women [2, 6, 7]. For example, in Sweden, the rise in smoking-related mortality in women and decline in smoking related mortality in men accounted for 40% of the narrowed GGLE between 1997 and 2016 [6].

While public health policies and quality healthcare play an important role in improving population health and longevity as well as tackling health disparities [8–11], less attention has been given to their contributions to the GGLE. The concept of “avoidable mortality” was introduced by Rutstein et al. [12] in the mid-1970s as “unnecessary untimely deaths” that would have been prevented by timely and effective healthcare intervention. It has been suggested as a potential indicator of the influences of public health policies and healthcare quality on population health and to identify potential areas for improvement [13]. A distinction is often made between avoidable causes that are *amenable* to secondary and tertiary prevention as well as medical interventions, and those avoidable causes that are *preventable* through public health policies and primary prevention [12]. In Sweden, avoidable mortality is one of the Swedish National Board of Health and Welfare monitoring indicators in accordance with Good Health Care [14]. Declining temporal trends in avoidable mortality have been reported in different countries including Sweden [15–20]. Moreover, these reductions in avoidable mortality were generally steeper than non-avoidable mortality and in men than in women. In addition, the absolute gains in LE due to avoidable mortality were larger for men than women in Europe [21–23], Asia [20], and New Zealand [24], while opposite was seen in Latin America [25]. While a recent study [2] investigated the impact of causes of deaths on the GGLE in Sweden between 1997 and 2014, this was not conducted by avoidable causes of death. In this study, we aimed to assess the contribution of healthcare to the GGLE in Sweden. Specifically, we aimed to 1) provide an updated comparison of temporal trends in avoidable and non-avoidable mortality during 1997–2018, and 2) quantify the contributions of avoidable causes to LE gain and the GGLE between 1997 and 2018.

## Method

### Data sources

We collected annual data on underlying causes of death by age and sex from the National Board of Health and Welfare’s Cause of Death Register [26] for the period 1997–2018. In this register, causes of death are coded according to the International Classification of Diseases, the 10th revision (ICD-10) since 1997. The Swedish National Cause of Death Register covers the deaths of all people registered in Sweden at the time of death, regardless of whether the death occurred inside or outside the country.

We followed the definition of avoidable mortality from the Office of National Statistics (ONS) in the UK [27] and divided these into four mutually exclusive categories: only amenable to healthcare, only preventable, amenable & preventable, and ischaemic heart disease (IHD) (Additional file 1). Three first categories were further divided into subcategories. While IHD belongs to amenable & preventable subgroup, we analysed this as a separate category because the large number of IHD deaths might mask trends and contributions of other amenable & preventable causes. Remaining causes of death were defined as non-avoidable causes. It should be noted that those avoidable causes that occurred beyond the age limit are considered as non- avoidable (e.g. IHD in 75+ age groups are non-avoidable). In addition, for some avoidable causes (mainly preventable causes) no age limit was considered. As our access to ICD-10 codes were limited to 3-digit, we did not count a few causes with four-digit ICD-10 code as avoidable causes.

### Analysis

We calculated age-standardized mortality rates for each cause by means of direct standardization with 5-year age groups (0–4, 5–9,…,85+) using the 2010 Organisation for Economic Cooperation and Development (OECD) population as standard. For trend analysis, we used the Joinpoint Regression Program version 4.7.0.0 from the Surveillance Research Program of the US National Cancer Institute (http://surveillance.cancer.gov/joinpoint). We selected heteroscedastic error option and the program used weighted least squares to handle this. We applied weighted Bayesian Information Criteria (BIC) option in the software to select the model (i.e. the number of joinpoints) that fit the data best and estimate an annual percentage change (APC) for each joinpoint from a log-linear model:

$$ Ln\left({ASMR}_y\right)={\beta}_0+{\beta}_1y $$

$$ APC=\left({e}^{\beta_1}-1\right)\times 100 $$

where ASMR_y_ shows age-standardized mortality rate at year y. A minimum number of 0 and a maximum number of 4 joinpoints were supplied and auto-correlated errors model (based on the data) was used. Then, the average annual percent change (AAPC) was computed as the weighted average of APCs to provide a summary measure of the trend for the whole time period. The 95% confidence interval for APC and AAPC were computed based on empirical quantile method. All analyses were performed separately for men and women.

We calculated LE at birth using abridged life tables [28] for the year 1997 and 2018. Then change in LE for each sex and the GGLE were decomposed into age– and cause–specific contributions using Arriaga’s method [29]. We used the Excel template of Auger et al. [30] to calculate LE and to perform the decomposition analysis.

## Results

During 1997–2018, 26.9 and 16.3% of all deaths were due to avoidable causes in men and women, respectively, with preventable causes constituting the largest proportion of avoidable causes in both sexes (12.7 and 7.1% of all deaths in men and women, respectively, Additional file 2). Across age groups, the share of avoidable causes from all deaths were greatest in those aged 20–24 years, with age groups of 0–4 years, 20–24 years, and 70–74 years had the largest proportions of amenable, preventable, and IHD, respectively (Additional file 3).

**Fig. 1:**
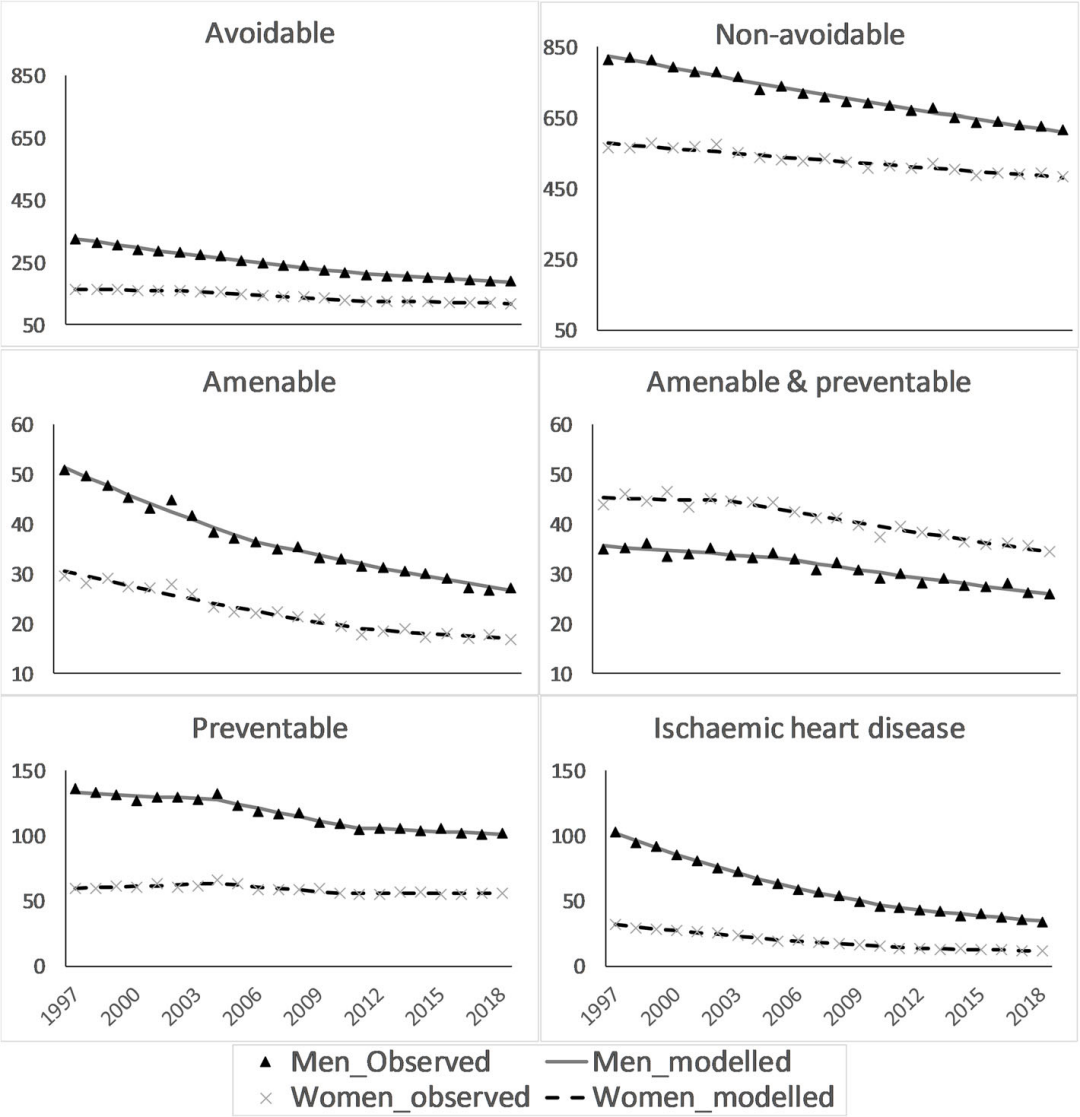
Observed and modelled (using joinpoint regression) age-standardized mortality rates (per 100,000 persons) during 1997–2018 in Sweden, by sex

Age-standardized mortality rates for all categories but amenable & preventable category were higher in men than in women (Fig. 1 and Additional files 4, 5, 6, 7). The joinpoint regression revealed that the average annual reductions in avoidable causes (2.6, 95% CI: 2.5, 2.7% in men; 1.6, 95% CI: 1.4, 1.7% in women) were more profound than reductions in non-avoidable causes (1.4, 95% CI: 1.3, 1.5% in men; 0.9, 95% CI: 0.7, 1.0% in women) (Tables 1 & 2). Across avoidable categories, while the average annual reductions in amenable, amenable &preventable, and IHD were comparable between men and women, more favourable reductions in preventable mortality was seen in men than in women. Although mortality for specific causes were generally declining, mortality in hypertensive diseases and accidental injury rose over the study period in both sexes. Moreover, only men not women observed reductions in mortality due to chronic obstructive pulmonary disorder (COPD).

**Table 1:** Changes in age-standardized mortality rates in men in Sweden, 1997–2018

Causes (ICD-10 codes)	Period	APC (95% CI)	AAPC (95% CI), 1997–2018
Amenable neoplasms (C54-C55, C62, C67, C73, C81, C91, D10-D36)	1997–2012	− 1.2 (− 1.8, − 0.3)	− 2.7 (− 3.3, − 2.1)
	2012–2018	− 6.2 (− 10.3, − 4.1)	
Hypertensive diseases (I10-I15)	1997–2018	2.5 (1.8, 3.4)	2.5 (1.8, 3.4)
Cerebrovascular diseases (I60-I69)	1997–2012	− 5.4 (− 6.1, − 5.1)	− 4.7 (− 5.1, − 4.5)
	2012–2018	− 3.1 (− 4.5, − 1.3)	
Pneumonia (J12-J18)	1997–2002	2.3 (0.5, 5.5)	−2.4 (− 2.9, − 1.9)
	2002–2006	− 17.4 (− 19.7, − 13.9)	
	2006–2010	8.3 (4.4, 11.6)	
	2010–2018	− 2.2 (− 4.2, − 1.2)	
*Total amenable*	1997–2006	− 3.8 (− 5.0, − 3.3)	−3.1 (− 3.3, − 2.9)
	2006–2018	− 2.6 (− 2.9, − 1.5)	
Preventable neoplasms (C00-C14, C15, C16, C22, C33-C34, C45)	1997–2004	− 1.2 (− 1.9, 0.5)	− 2.0 (− 2.3, − 1.8)
	2004–2018	−2.4 (− 3.3, − 2.2)	
Alcohol related and illicit drug use diseases (F10, K70, K73, K74, F11-F16, F18-F19)	1997–2018	− 3.4 (− 4.1, − 2.7)	−3.4 (− 4.1, − 2.7)
Transport accidents (V01-V99)	1997–2001	3.1 (− 2.8, 9.0)	− 3.2 (− 4.4, − 2.3)
	2001–2014	− 6.7 (− 12.9, − 6.1)	
	2014–2018	2.4 (− 4.8, 7.1)	
Accidental injury (W00-X59)	1997–2003	3.4 (1.3, 7.7)	1.1 (0.5, 1.7)
	2003–2018	0.2 (− 1.4, 0.7)	
Suicide and self-inflicted injuries (X60-X84, Y10-Y34)	1997–2018	− 0.8 (− 1.2, − 0.4)	− 0.8 (− 1.2, − 0.4)
*Total preventable*	1997–2004	− 0.6 (− 1.2, 0.5)	− 1.3 (− 1.5, − 1.1)
	2004–2011	− 2.6 (− 4.1, − 2.1)	
	2011–2018	− 0.6 (− 1.2, 0.7)	
Amenable & preventable neoplasms (C18-C21, C43, C50, C53)	1997–2009	− 0.5 (− 0.9, 0.2)	−1.2 (− 1.5, − 1.0)
	2009–2018	− 2.2 (− 3.3, − 1.6)	
Chronic obstructive pulmonary disorder (J40-J44)	1997–2003	− 1.1 (− 2.1, 1.3)	− 2.2 (− 2.5, − 1.8)
	2003–2010	− 4.0 (− 6.2, − 3.2)	
	2010–2018	− 1.4 (− 2.3, 0.5)	
*Total amenable & preventable*	1997–2005	− 0.8 (− 1.3, 0.7)	− 1.5 (− 1.7, − 1.2)
	2005–2018	−1.9 (− 2.7, − 1.6)	
*Ischaemic heart disease (I20-I25)*	1997–2011	− 5.8 (− 6.0, − 5.6)	−5.0 (− 5.2, − 4.9)
	2011–2018	− 3.5 (− 4.2, − 2.4)	
**Total avoidable**	1997–2011	− 3.0 (− 3.4, − 2.8)	−2.6 (− 2.7, − 2.5)
	2011–2018	− 1.8 (− 2.4, − 0.8)	
**Non-avoidable**	1997–2018	− 1.4 (− 1.5, − 1.3)	−1.4 (− 1.5, − 1.3)

*APC* Annual Percentage Change, *AAPC* Average Annual Percentage Change, *CI* Confidence Interval

**Table 2:** Changes in age-standardized mortality rates in women in Sweden, 1997–2018

Causes (ICD-10 codes)	Period	APC (95% CI)	AAPC (95% CI), 1997–2018
Amenable neoplasms (C54-C55, C62, C67, C73, C81, C91, D10-D36)	1997–2018	−2.1 (− 2.8, − 1.3)	−2.1 (− 2.8, − 1.3)
Hypertensive diseases (I10-I15)	1997–2018	2.4 (1.3, 3.7)	2.4 (1.3, 3.7)
Cerebrovascular diseases (I60-I69)	1997–2002	−2.4 (− 4.0, 0.3)	−4.2 (− 4.6, − 3.8)
	2002–2010	− 6.3 (− 9.2, − 5.5)	
	2010–2018	−3.3 (− 4.5,-0.5)	
Pneumonia (J12-J18)	1997–2006	−7.4 (− 13.2, − 4.6)	−2.7 (− 3.7, − 1.6)
	2006–2018	1.0 (− 1.1, 6.0)	
*Total amenable*	1997–2011	−3.4 (− 5.4, − 2.4)	−2.8 (− 3.2, − 2.4)
	2011–2018	−1.6 (− 3.1, 0.9)	
Preventable neoplasms (C00-C14, C15, C16, C22, C33-C34, C45)	1997–2005	1.6 (0.9, 2.9)	− 0.3 (− 0.5, 0.0)
	2005–2018	−1.4 (− 1.8, − 1.0)	
Alcohol related and illicit drug use diseases (F10, K70, K73, K74, F11-F16, F18-F19)	1997–2011	− 2.7 (− 5.9, − 1.7)	− 1.4 (− 2.2, − 0.8)
	2011–2018	1.1 (− 1.5, 5.7)	
Transport accidents (V01-V99)	1997–2007	− 2.8 (− 4.2, 0.1)	−4.3 (− 5.2, − 3.4)
	2007–2011	−13.7 (− 17.4, − 7.8)	
	2011–2018	− 0.8 (− 4.4, 6.9)	
Accidental injury (W00-X59)	1997–2018	0.5 (− 0.1, 1.2)	0.5 (− 0.1, 1.2)
Suicide and self-inflicted injuries (X60-X84, Y10-Y34)	1997–2018	− 0.1 (− 0.7, 0.5)	−0.1 (− 0.7, 0.5)
*Total preventable*	1997–2004	1.0 (0.2, 2.6)	−0.3 (− 0.5, − 0.0)
	2004–2010	− 2.1 (− 3.8, − 1.2)	
	2010–2018	−0.1 (− 0.8, 1.5)	
Amenable & preventable neoplasms (C18-C21, C43, C50, C53)	1997–2005	−0.9 (− 1.3, 0.1)	−1.6 (− 1.8, − 1.4)
	2005–2018	− 2.0 (− 2.5, − 1.8)	
Chronic obstructive pulmonary disorder (J40-J44)	1997–2002	4.8 (2.5, 8.8)	0.5 (0.1, 1.1)
	2002–2008	−2.4 (− 4.9, − 1.0)	
	2008–2018	0.1 (− 0.6, 2.3)	
*Total amenable & preventable*	1997–2003	− 0.2 (− 1.0, 1.4)	− 1.3 (− 1.5, − 1.1)
	2003–2018	−1.7 (− 2.1, − 1.5)	
*Ischaemic heart disease (I20-I25)*	1997–2012	− 5.5 (− 6.9, − 5.1)	−4.8 (− 5.3, − 4.6)
	2012–2018	−3.1 (− 4.9, − 0.8)	
**Total avoidable**	1997–2003	− 0.8 (− 1.3, 0.0)	− 1.6 (− 1.7. -1.4)
	2003–2011	− 2.7 (− 3.8, − 2.3)	
	2011–2018	− 0.9 (− 1.4, 0.0)	
**Non-avoidable**	1997–2018	− 0.9 (− 1.0, − 0.7)	−0.9 (− 1.0, − 0.7)

*APC* Annual Percentage Change, *AAPC* Average Annual Percentage Change, *CI* Confidence Interval

Between 1997 and 2018, LE in men rose by 4.1 years (from 72.8 to 76.9 years), of which 2.4 (59.3%) years were attributed to avoidable deaths (Table 3, detailed results are presented in Table 1 in Additional file 8). Among women, there was 2.3 years gain in LE at the same period (from 78.0 to 80.3 years), of which 1.0 (45.6%) years were attributed to avoidable deaths (Table 2 in Additional file 8). While both sexes experienced decline in LE from accidental injury and hypertensive diseases, only women seen decreases in LE from suicide and self-inflicted injuries as well as COPD. Age group 70–74 years had the greatest contributions to the gain in LE from avoidable causes in both sexes. For non-avoidable causes, the age group 80–84 years and 75–79 years had the greatest contributions to the LE gain among women and men, respectively. The largest contributions to decline in LE were seen among men 25–29 years from accidental injury and women 70–74 years from preventable neoplasms.

**Table 3:** Contribution of causes of death to changes in life expectancy and gender gap in life expectancy in Sweden between 1997 and 2018

Causes (ICD-10 codes)	Women LE, 1997–2018, years (%)	Men LE, 1997–2018, years (%)	Gender gap in LE, 1997, years (%)	Gender gap in LE, 2018, years (%)	Change in gender gap in LE, years (%)
Amenable neoplasms (C54-C55, C62, C67, C73, C81, C91, D10-D36)	0.02 (0.7)	.05 (1.3)	0.06 (1.1)	0.02 (0.6)	−0.04 (− 2.0)
Hypertensive diseases (I10-I15)	− 0.01 (− 0.3)	−0.01 (− 0.1)	0.02 (0.4)	0.02 (0.7)	0.00 (0.1)
Cerebrovascular diseases (I60-I69)	0.19 (8.3)	0.25 (6.2)	0.17 (3.3)	.08 (2.4)	−0.09 (− 5.1)
Pneumonia (J12-J18)	0.03 (1.4)	0.03 (0.6)	0.03 (0.5)	0.03 (1.0)	0.00 (0.3)
*Total amenable*	*0.30 (13.2)*	*0.46 (11.1)*	*0.41 (7.8)*	*0.23 (6.7)*	*−0.18 (−9.8)*
Preventable neoplasms (C00-C14, C15, C16, C22, C33-C34, C45)	0.06 (2.4)	0.28 (6.9)	0.36 (6.9)	0.14 (4.0)	−0.22 (− 12.0)
Alcohol related and illicit drug use diseases (F10, K70, K73, K74, F11-F16, F18-F19)	0.02 (0.8)	0.18 (4.4)	0.27 (5.2)	0.11 (3.4)	−0.16 (− 8.4)
Transport accidents (V01-V99)	0.06 (2.6)	0.15 (3.7)	0.19 (3.7)	0.10 (2.9)	−0.09 (− 5.1)
Accidental injury (W00-X59)	−0.06 (−2.7)	− 0.11 (− 2.7)	0.25 (4.7)	0.35 (10.5)	0.11 (5.9)
Suicide and self-inflicted injuries (X60-X84, Y10-Y34)	−0.01 (− 0.7)	0.08 (2.1)	0.38 (7.3)	0.30 (8.9)	−0.08 (−4.2)
*Total preventable*	*0.12 (5.4)*	*0.68 (16.5)*	*1.56 (30.0)*	*1.10 (32.6)*	*−0.47 (−25.2)*
Amenable & preventable neoplasms (C18-C21, C43, C50, C53)	0.23 (10.1)	0.06 (1.4)	−0.35 (−6.7)	− 0.23 (− 6.7)	0.12 (6.6)
Chronic obstructive pulmonary disorder (J40-J44)	− 0.01 (− 0.5)	0.04 (1.0)	0.02 (0.5)	− 0.03 (−1.0)	−0.06 (−3.1)
*Total amenable & preventable*	*0.22 (9.9)*	*0.16 (4.0)*	*−0.22 (−4.1)*	*− 0.20 (−5.9)*	*0.02 (0.9)*
*Ischaemic heart disease (I20-I25)*	*0.39 (17.2)*	*1.14 (27.7)*	*1.25 (23.9)*	*0.44 (13.1)*	*−0.81(−43.6)*
**Total avoidable**	1.03 (45.6)	2.44 (59.3)	3.00 (57.6)	1.57 (46.5)	−1.44 (− 77.7)
**Non-avoidable**	1.23 (54.4)	1.67 (40.7)	2.21 (42.4)	1.80 (53.5)	−0.41 (−22.3)
**Total**	2.26 (100)	4.11 (100)	5.22 (100)	3.37 (100)	−1.85 (100)

*LE* Life expectancy

Negative values in columns (2) and (3) show a negative contribution to LE. Negative values in columns (4) and (5) indicate LE disadvantage in women. Negative values in column (6) indicate narrowing the gender gap in LE and positive values indicate widening the gender gap in LE

**Fig. 2:**
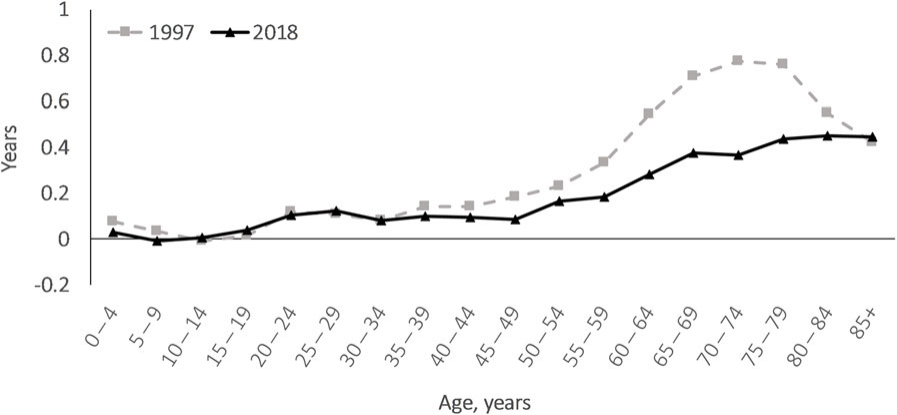
Age-specific contributions to the gender gap in life expectancy in Sweden in 1997 and 2018

**Fig. 3:**
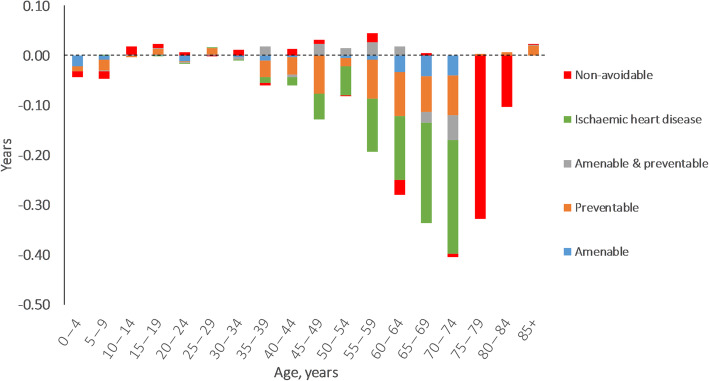
Age- and cause-specific contributions to the change of gender gap in life expectancy in Sweden between 1997 and 2018. Positive values indicate widening gender gap in life expectancy and negative values indicate narrowing gender gap in life expectancy

In 1997, women lived 5.2 years longer than men and avoidable causes accounted for 3.0 years (57.6%) of this (Table 3 in Additional file 8). The corresponding GGLE was 3.4 years in 2018, of which avoidable causes were responsible for 1.6 years (46.5%) (Table 4 in Additional file 8). While the contributions of age groups of 0–64 years to the GGLE remained stable between 1997 and 2018 (38.4% in 1997 and 38.3% in 2018), it declined for age groups of 65–79 years (from 43.0 to 35.0%) and rose for those aged≥80 years (from 18.6 to 26.6%) (Fig. 2). Among avoidable causes, preventable causes had the greatest contributions to the GGLE. Contributions from accidental injury to the GGLE rose substantially between 1997 and 2018 (from 4.7 to 10.5%). Moreover, the LE advantage of women from COPD reversed between 1997 and 2018. IHD had the greatest contribution to the narrowing GGLE (0.8 years) followed by preventable causes (0.5 years, with preventable neoplasms and alcohol- & drug-related deaths accounted for 80% of this) and non-avoidable causes (0.4 years) (Table 3 & Fig. 3). Cerebrovascular diseases accounted for more than half of the contributions of amenable causes to the narrowing GGLE. The age groups of 60–79 years accounted for 71.7% of the narrowed GGLE (Fig. 3). Hypertensive diseases, pneumonia, incidental injury and amenable & preventable neoplasms (including breast cancer) contributed to widening the GGLE, even though the contributions from hypertensive diseases and pneumonia were negligible (0.01 years combined).

## Discussion

This study documented more profound reductions in avoidable mortality than in non-avoidable mortality over recent two decades in Sweden. However, mortality for several avoidable causes including hypertensive diseases and accidental injury rose over time. Avoidable causes accounted for a significant proportion of LE gain with a larger contribution in men than in women. In addition, over the half of LE gain from avoidable causes were resulted from declined mortality in people aged 60–74 years. The GGLE has narrowed by 1.9 years and avoidable causes accounted for about three quarters of this. Among avoidable causes, IHD had the largest contributions to gains and the narrowing GGLE.

Our results showed that observed decline in avoidable mortality in Sweden during 1971─1996 [18] have persisted in recent two decades. Despite using a somewhat different definition of amenable conditions, a recent study reported average annual reductions of 3.5 and 2.9% in amenable mortality for men and women, respectively, in Sweden over 2000–2013 [31] which were comparable to our estimates. Consistent with previous studies, we also found more profound reductions in avoidable causes compared with non-avoidable causes [20, 31, 32] which translated into greater contribution of avoidable than non-avoidable deaths on LE gain between 1997 and 2018. These improvements have been attributed to diagnostic and therapeutic innovation (especially for cardiovascular disease), improvement in quality of care, and reduction in incidence of underlying diseases and their risk factors [33, 34].

The observed steeper decline in amenable causes compared with preventable causes is in line with previous studies [20, 22]. While further investigation is required to explore underlying mechanisms, this finding highlight the need for strengthening of inter-sectoral public health policies aimed at behavioural changes. In particular, LE loss from accidental injury in men aged 25–39 years and from smoking-related mortality (e.g., malignant neoplasm of bronchus and lung, COPD) in women 70–74 years require urgent attention. Moreover, hypertensive diseases were another category with worrying rising trends in both sexes which despite being defined as amenable cause can also benefit from lifestyle behaviours including smoking and obesity. Substantial reductions in IHD and cerebrovascular diseases highlight potential contributions of advances in diagnostic and therapeutic innovation including treatment of hypertension, intensive management of acute stroke (e.g. CT-scan, thrombolytic therapy), and improvement in the management of myocardial infarction (e.g. β-blockers, ACE-inhibitors) [33, 35].

Notwithstanding encouraging reductions in avoidable mortality, a slowdown in the declining trends of avoidable mortality in both sexes during 2010s is of concern. A recent study suggested that European countries with private provision experienced a slowdown in the decline of amenable mortality rates [36]. During 2007–2010, Swedish primary healthcare underwent market-oriented reforms involving free choice of provider and freedom of establishment for private primary care providers [37] and this might partially explain the slower decline in most recent years in Sweden. It should be noted that while we observed a slowdown in amenable mortality among men already in 2006, Gianino et al. [36] included IHD death as amenable in their study which had a slowdown in 2011–2012 in both sexes in this study. Furthermore, while Swedish economy and health system performed well in response to the global financial crisis in 2008 [38], we cannot rule out the potential role of the crisis in observed slowdown of the declining trends of avoidable mortality, especially on preventable deaths through its impact on people’s lifestyle and mental health [39, 40].

The greater improvements in LE for men than for women and, hence, narrowing the GGLE over recent decades is well-documented in Sweden and other developed countries [1, 2, 41]. Consistent with recent findings in Sweden [2], we found that the decreased mortality from IHD, particularly among those aged 65–74 years, had the greatest contribution to the narrowed GGLE. The declining gender gap in lifestyles including smoking and alcohol drinking has been suggested as an important contributor of the narrowing GGLE [2, 6, 7]. Indeed, two recent studies [2, 6] documented rise in smoking-related mortality in women and decline in men in Sweden, which is consistent with more favourable reductions in alcohol- and smoking-related death in men than in women observed in this study. It should be noted that smoking and alcohol consumption are also associated with increased risk of IHD mortality [35, 42]. The narrowing GGLE due to more favourable behavioural changes in men than in women is to some extent a consequence of the fact that men generally took up unhealthy lifestyles behaviours years (even decades) before women and hence were being subject to earlier preventive measures [43].

It is also argued that medical advancement, especially those for cardiovascular diseases, might have benefited men more than women [44, 45]. However, since declines in mortality from amenable causes and IHD were comparable between men and women, this is unlikely to account for the observed narrowed GGLE in this study. In fact, the comparability in mortality trends implies that the narrowing GGLE is partially due to gender differences in the age pattern of mortality, that is the same reduction in mortality yields larger gain in LE for men than for women due to a less dispersed age distribution of death among women [44]. Moreover, as mortality rates were generally higher in men than in women, the same proportional decline in mortality might yield greater contributions, in absolute terms, to the gains in LE among men than women.

In line with previous research [2, 46], the contributions to the GGLE from oldest age groups (80+) rose over time which was expected considering increases in LE. Consequently, the age groups < 60 years had the greatest contributions to the GGLE in 2018 whereas in 1997 the age groups 65–79 had the largest contributions. Greater reductions in mortality from amenable & preventable neoplasms in women contributed to widening the GGLE in those aged < 65 years and this is partially due to the presence of breast cancer among these causes.

Some limitations of the current study should be highlighted. The data used in our study were obtained from death certificates which are known to suffer from coding errors, diagnostic inaccuracy, and underreporting. In particular, we cannot rule out the potential effects of changes in coding practices over time on our findings. However, the magnitude of these problems are unlikely to be considerable considering the good-quality of Swedish vital statistics system [47]. Similar to common practice in cause-of-death analysis, we relied on underlying cause of death which might underestimates the involvement of the chronic conditions, especially among older people suffering from several comorbidities [48]. The attribution of causes of death to “avoidable” and its different subcategories requires some degree of judgment (e.g., about the effectiveness of health policies and medical interventions, the choice of upper age limit) [13]. Moreover, the concept is essentially time-dependent as it will change with development of new medical technologies and health policies over time [13]. There are also different lists of avoidable causes and selection of the one might have substantial influence on a study’s findings [49]. All these issues limit the between-study comparability and also generalizability of our findings to other settings. Furthermore, avoidable mortality doesn’t take into account the underlying prevalence of diseases and their severity as well as the effects of health policies and medical interventions on quality of life [49]. These drawbacks imply that avoidable mortality is an incomplete measure of the effectiveness of health policies and quality healthcare. Investigating the age and cause contributions from cohort perspective (compared with period perspective in our study) can provide more insights on the patterns of GGLE and is subject for future research. We used the Arriaga’s method for decomposition which may underestimate the contributions for causes of death that occur mainly at older ages [50]. It should be also noted that this is a descriptive epidemiological study and all given explanations for mortality trends and associated causes are speculative.

## Conclusion

Our findings revealed more profound reductions in avoidable mortality than non-avoidable mortality during recent two decades in Sweden. These reductions translated into substantial contributions of avoidable causes into rising LE and the narrowing GGLE in Sweden. Despite these encouraging observations, rises in mortality from hypertensive diseases and accidental injury in both sexes as well as COPD in women are of concern. Our findings highlight the need for further improvements in preventive measures and inter-sectoral health policies especially among women.

## Supplementary Information

**Additional file 1:.** List of avoidable causes of death according to the UK Office of National Statistics.

**Additional file 2:.** The distribution of causes of death from 1997 to 2018 in Sweden, by sex.

**Additional file 3:.** The distribution of causes of death during 1997–2018 in Sweden, by age.

**Additional file 4:.** Observed and modelled (using joinpoint regression) age-standardized mortality rates (per 100,000 persons) for subcategories of amenable causes during 1997–2018 in Sweden, by sex.

**Additional file 5:.** Observed and modelled (using joinpoint regression) age-standardized mortality rates (per 100,000 persons) for subcategories of preventable causes during 1997–2018 in Sweden, by sex.

**Additional file 6:.** Observed and modelled (using joinpoint regression) age-standardized mortality rates (per 100,000 persons) for subcategories of amenable & preventable causes during 1997–2018 in Sweden, by sex.

**Additional file 7:.** Observed age-standardized mortality rates (per 100,000 persons) for years 1997, 2010, and 2018 in Sweden, by sex.

**Additional file 8:.** Detailed age- and cause-specific contributions to changes and gender gap in life expectancy.

## Data Availability

The dataset supporting the conclusions of this article is available in the Statistical databases of the National Board of Health and Welfare website (https://sdb.socialstyrelsen.se/if_dor/val_eng.aspx).

## Abbreviations

APC: Annual percentage change
AAPC: Average annual percentage change
CI: Confidence interval
GGLE: Gender gap in life expectancy
ICD-10: International classification of diseases, the 10th revision
IHD: Ischaemic heart disease
LE: Life expectancy
