# Supplementary material for: Avoidable deaths in Sweden, 1997–2018: temporal trend and the contribution to the gender gap in life expectancy
Source: BMC Public Health. 2021 Mar 17;21:519. doi: 10.1186/s12889-021-10567-5 (PMC7968161; doi:10.1186/s12889-021-10567-5)
Supplement: Supplementary file 1 — Additional file 1:. List of avoidable causes of death according to the UK Office of National Statistics. [file 12889_2021_10567_MOESM1_ESM.pdf]

List of avoidable causes of death according to the UK Office of National Statistics

Category	Cause	ICD-10 codes	Age limit
**Amenable to healthcare**	Selected invasive bacterial and protozoal infections	A38-A41, A46, B50-B54, G00, G03, J02, L03	0-74
	Intestinal infections	A00-A09	0-14
	Malignant neoplasm of unspecified parts of uterus and body of uterus	C54-C55	0-44
	Malignant neoplasm of testis	C62	0-74
	Malignant neoplasm of bladder	C67	0-74
	Malignant neoplasm of thyroid gland	C73	0-74
	Hodgkin's disease	C81	0-74
	Leukaemia	C91	0-44
	Benign neoplasms	D10-D36	0-74
	Diseases of the Thyroid	E00-E07	0-74
	Epilepsy and status epilepticus	G40-G41	0-74
	Rheumatic and other valvular heart disease	I01-I09	0-74
	Hypertensive diseases	I10-I15	0-74
	Cerebrovascular diseases	I60-I69	0-74
	Pneumonia	J12-J18	0-74
	Asthma	J45-J46	0-74
	Selected respiratory diseases	J00-J01, J03-J06, J20-J22, J30-J39	0-14
	Gastric and duodenal ulcer	K25-K28	0-74
	Acute abdomen, appendicitis, intestinal obstruction, cholecystitis/lithiasis, pancreatitis, hernia	K35-K38, K40-K46, K80-K83, K85-K86	0-74
	Nephritis and nephrosis	N00-N07, N17-N19, N25-N27	0-74
	Obstructive uropathy and prostatic hyperplasia	N13, N20-N21, N35, N40	0-74
	Complications of perinatal period	P00-P96, A33	All
	Congenital malformations of the circulatory system	Q20-Q28	0-74
**Preventable**	Rubella	B06	0-14
	Malignant neoplasm of lip, oral cavity and pharynx	C00-C14	0-74
	Malignant neoplasm of oesophagus	C15	0-74
	Malignant neoplasm of stomach	C16	0-74
	Malignant neoplasm of liver	C22	0-74
	Malignant neoplasm of trachea, bronchus and lung	C33-C34	0-74
	Mesothelioma	C45	0-74
	Alcohol related diseases, excluding external causes	F10, K70, K73, K74	0-74
	Illicit drug use disorders	F11-F16, F18-F19	0-74
	DVT with pulmonary embolism	I26, I80	0-74
	Aortic aneurysm and dissection	I71	0-74
	Spina Bifida	Q05	0-74
	Transport accidents	V01-V99	All
	Accidental injury	W00-X59	All
	Suicide and self-inflicted injuries	X60-X84, Y10-Y34	All
	Homicide/Assault	X85-Y09	All
**Amenable & preventable**	Tuberculosis	A15-A19, B90	0-74
	Pertussis (whooping cough)	A37	0-14
	Measles	B05	0-14
	Other infections (Diphtheria, Tetanus, Poliomyelitis and Varicella)	A35, A36, A80, B01	0-19
	HIV/AIDS	B20-B24	All
	Malignant neoplasm of colon and rectum	C18-C21	0-74
	Malignant melanoma of skin	C43	0-74
	Malignant neoplasm of breast	C50	0-74
	Malignant neoplasm of cervix uteri	C53	0-74
	Diabetes mellitus	E10-E14	0-74
	Influenza (including swine flu)	J09-J11	0-74
	Chronic obstructive pulmonary disorder	J40-J44	0-74
	Misadventures to patients during surgical and medical care	Y60-Y69, Y83-Y84	All
**Avoidable ischaemic heart disease**	Ischaemic heart disease	I20-I25	0-74
