# Supplementary figures and images for: Avoidable deaths in Sweden, 1997–2018: temporal trend and the contribution to the gender gap in life expectancy
Source: BMC Public Health. 2021 Mar 17;21:519. doi: 10.1186/s12889-021-10567-5 (PMC7968161; doi:10.1186/s12889-021-10567-5)

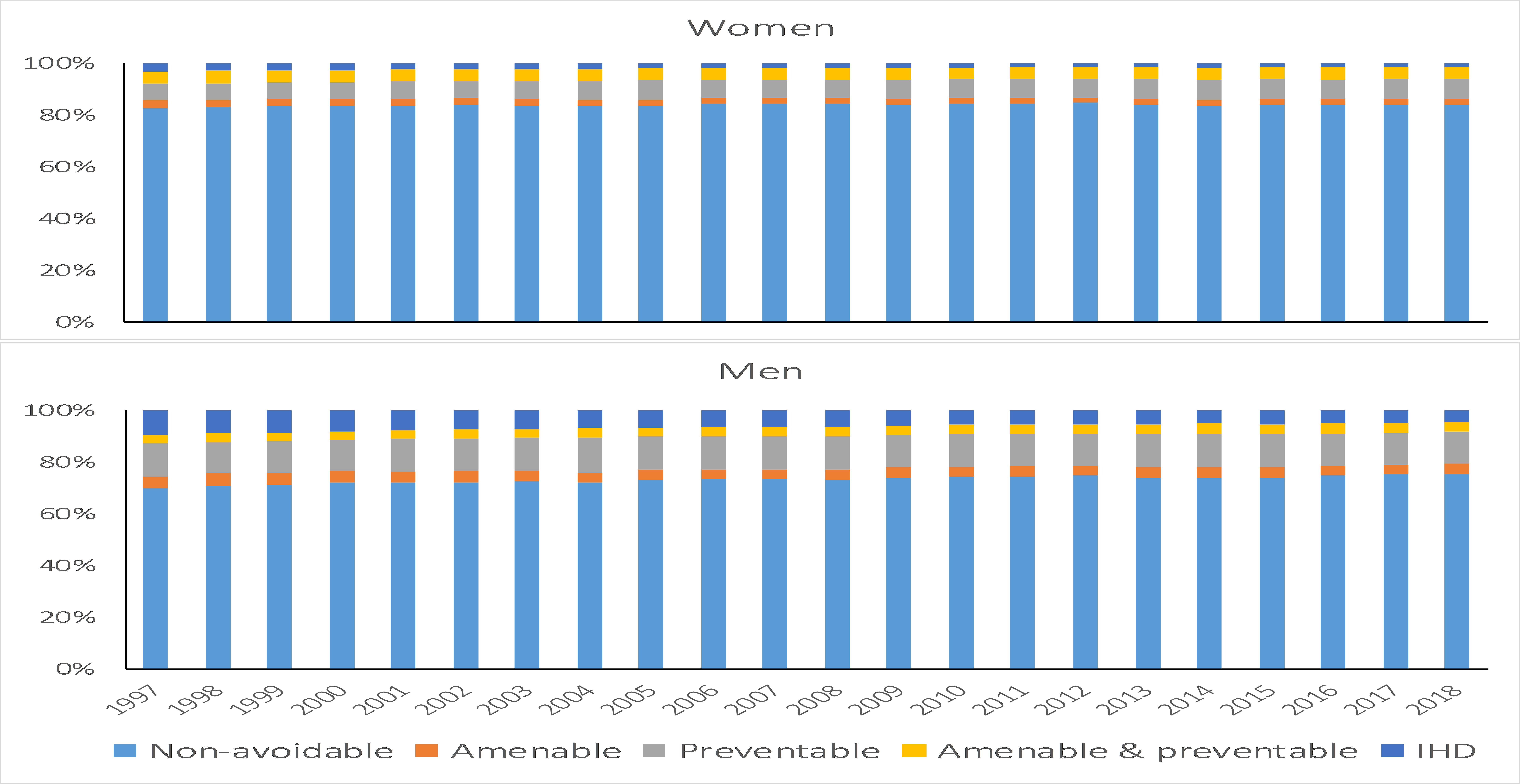

Supplement: Supplementary file 2 — Additional file 2:. The distribution of causes of death from 1997 to 2018 in Sweden, by sex. [file 12889_2021_10567_MOESM2_ESM.tif]

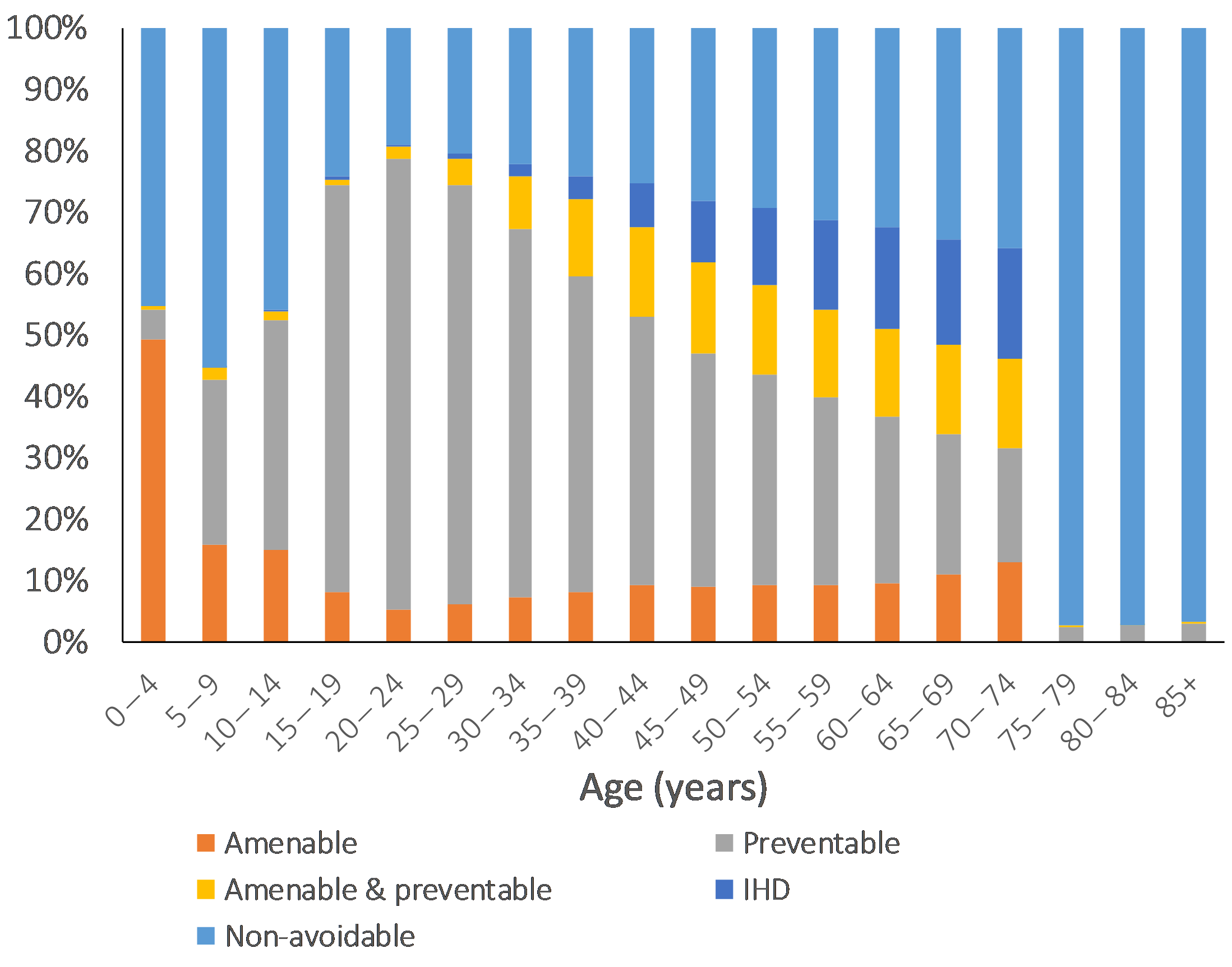

Supplement: Supplementary file 3 — Additional file 3:. The distribution of causes of death during 1997–2018 in Sweden, by age. [file 12889_2021_10567_MOESM3_ESM.tif]

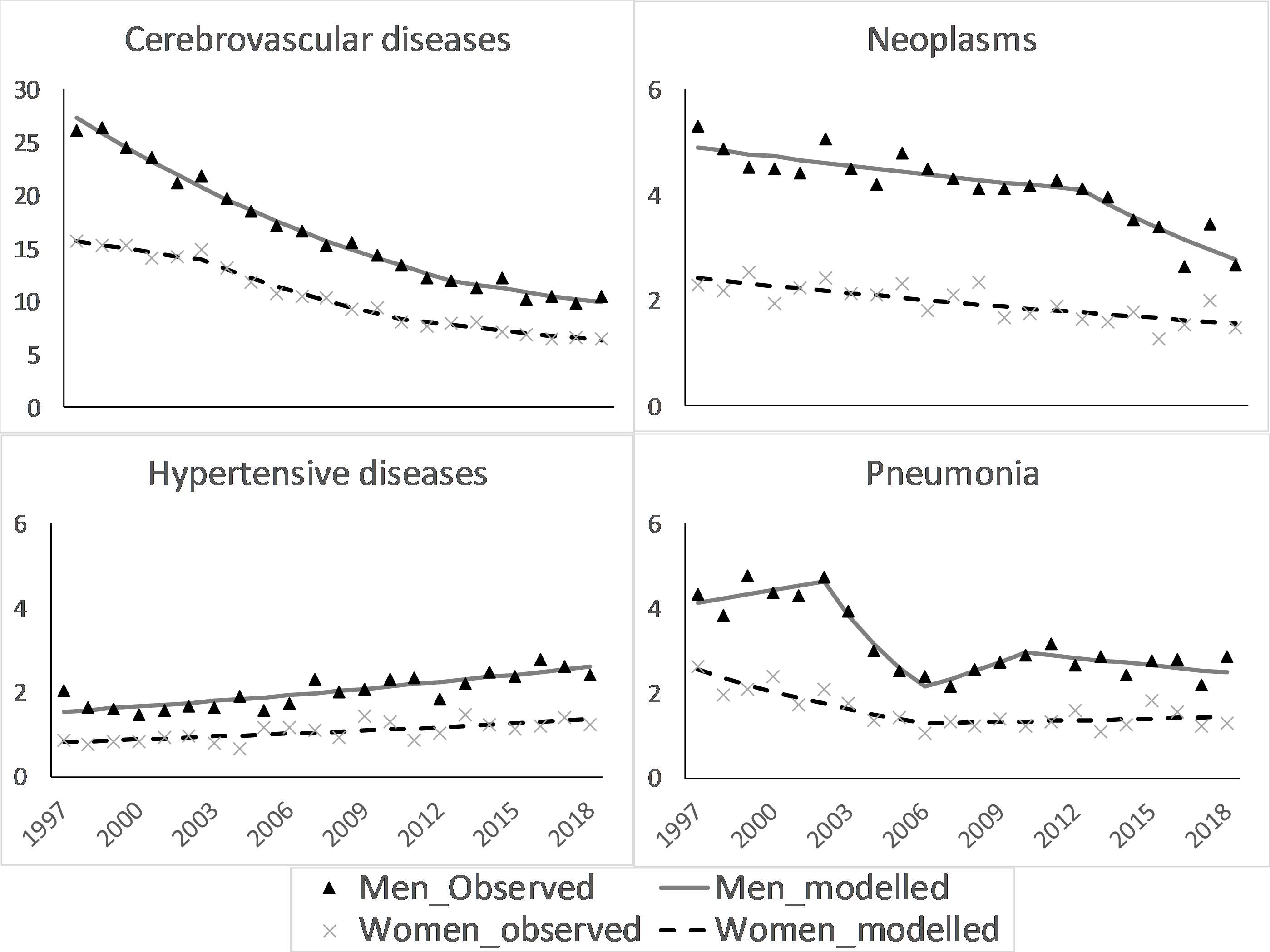

Supplement: Supplementary file 4 — Additional file 4:. Observed and modelled (using joinpoint regression) age-standardized mortality rates (per 100,000 persons) for subcategories of amenable causes during 1997–2018 in Sweden, by sex. [file 12889_2021_10567_MOESM4_ESM.tif]

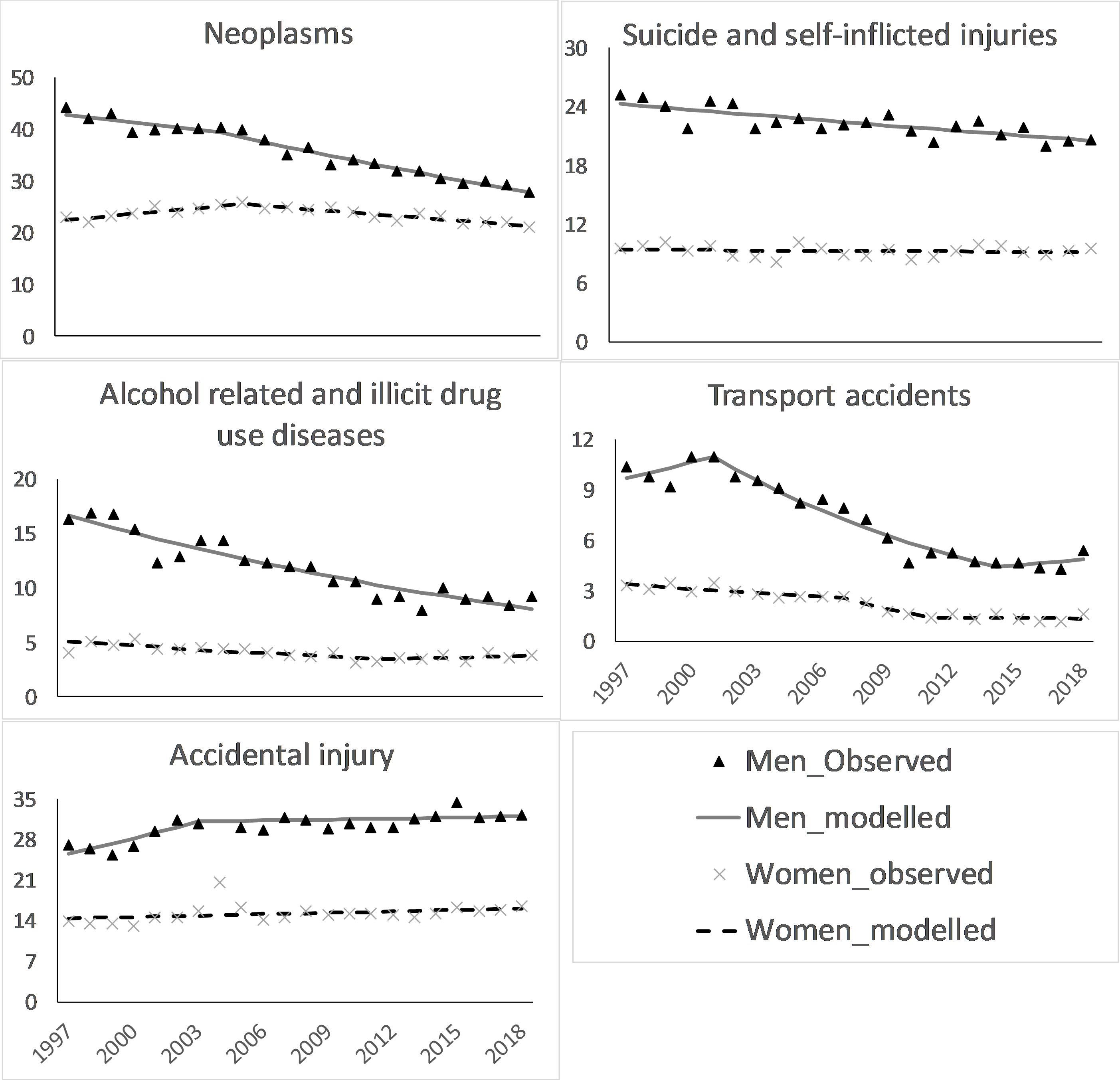

Supplement: Supplementary file 5 — Additional file 5:. Observed and modelled (using joinpoint regression) age-standardized mortality rates (per 100,000 persons) for subcategories of preventable causes during 1997–2018 in Sweden, by sex. [file 12889_2021_10567_MOESM5_ESM.tif]

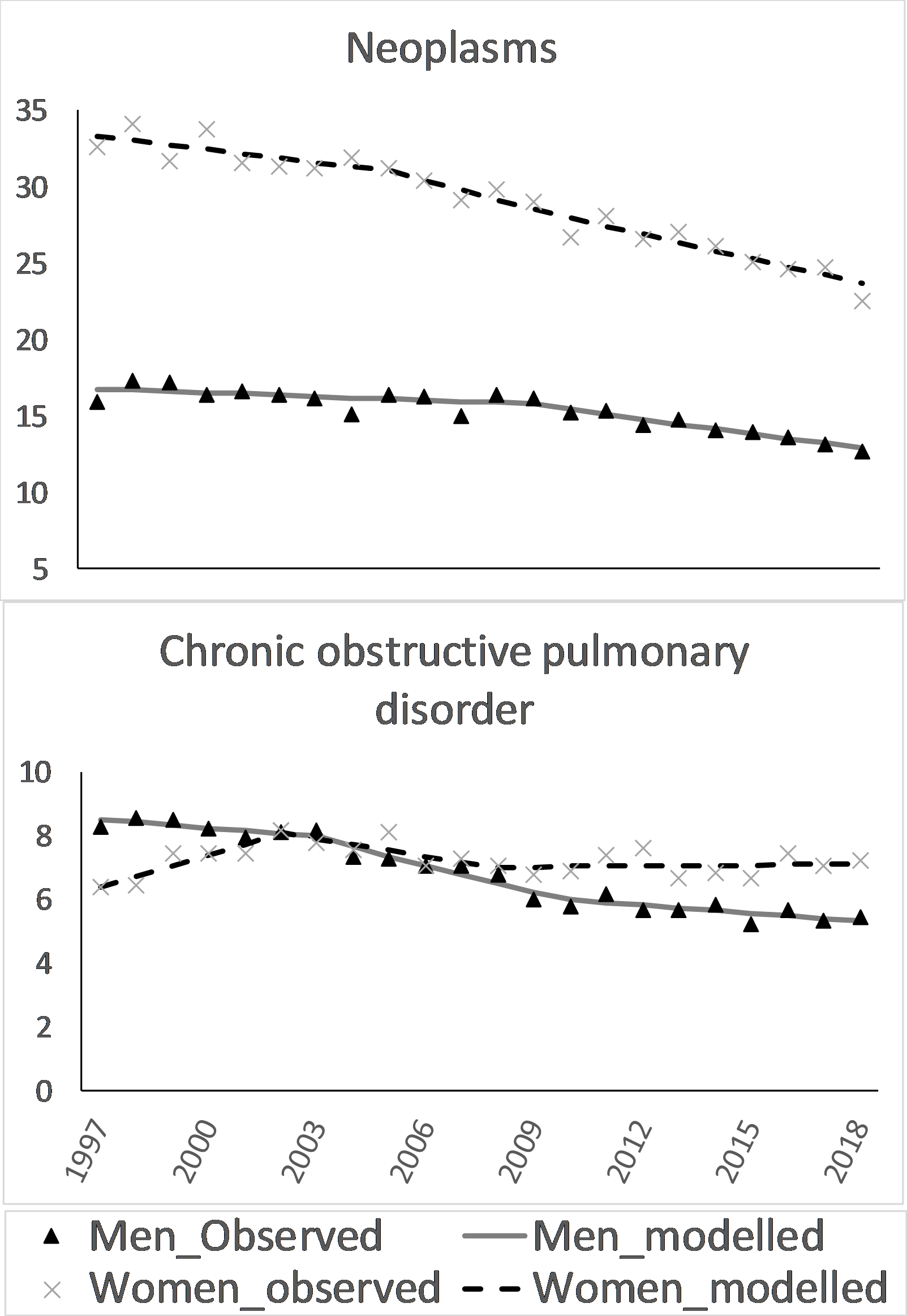

Supplement: Supplementary file 6 — Additional file 6:. Observed and modelled (using joinpoint regression) age-standardized mortality rates (per 100,000 persons) for subcategories of amenable & preventable causes during 1997–2018 in Sweden, by sex. [file 12889_2021_10567_MOESM6_ESM.tif]
