# Supplementary material for: Avoidable deaths in Sweden, 1997–2018: temporal trend and the contribution to the gender gap in life expectancy
Source: BMC Public Health. 2021 Mar 17;21:519. doi: 10.1186/s12889-021-10567-5 (PMC7968161; doi:10.1186/s12889-021-10567-5)
Supplement: Supplementary file 7 — Additional file 7:. Observed age-standardized mortality rates (per 100,000 persons) for years 1997, 2010, and 2018 in Sweden, by sex. [file 12889_2021_10567_MOESM7_ESM.pdf]

Additional file 7. Observed age-standardized mortality rates (per 100,000 persons) for years 1997, 2010, and 2018 in Sweden, by sex.

Causes (ICD-10 codes)	Men			Women		
	1997	2010	2018	1997	2010	2018
Amenable neoplasms (C54-C55, C62, C67, C73, C81, C91, D10-D36)	5.3	4.2	2.7	2.3	1.8	1.5
Hypertensive diseases (I10-I15)	2.0	2.3	2.4	0.9	1.3	1.2
Cerebrovascular diseases (I60-I69)	26.2	13.4	10.5	15.7	8.1	6.5
Pneumonia (J12-J18)	4.3	2.9	2.9	2.6	1.2	1.3
Total amenable	50.9	33.1	27.3	29.7	19.4	16.8
Preventable neoplasms (C00-C14, C15, C16, C22, C33-C34, C45)	44.2	34.0	27.8	22.9	24.0	21.0
Alcohol related and illicit drug use diseases (F10, K70, K73, K74, F11-F16, F18-F19)	16.3	10.6	9.2	4.1	3.1	3.8
Transport accidents (V01-V99)	10.4	4.7	5.4	3.3	1.6	1.6
Accidental injury (W00-X59)	27.1	30.8	32.3	13.9	15.2	16.5
Suicide and self-inflicted injuries (X60-X84, Y10-Y34)	25.2	21.5	20.7	9.5	8.4	9.5
Total preventable	136.2	109.1	102.1	60.0	55.5	55.7
Amenable & preventable neoplasms (C18-C21, C43, C50, C53)	15.9	15.2	12.7	32.6	26.7	22.5
Chronic obstructive pulmonary disorder (J40-J44)	8.3	5.8	5.4	6.4	6.9	7.2
Total amenable & preventable	35.0	29.1	25.9	43.9	37.4	34.4
Ischaemic heart disease (I20-I25)	103.1	46.1	33.4	31.6	15.5	11.6
Total avoidable	325.3	217.4	188.7	165.2	127.8	118.5
Non-avoidable	813.3	686.0	618.0	567.6	513.7	485.0
